# Human mammary fibroblasts stimulate invasion of breast cancer cells in a three-dimensional culture and increase stroma development in mouse xenografts

**DOI:** 10.1186/1471-2407-10-444

**Published:** 2010-08-19

**Authors:** Charlotta J Olsen, José Moreira, Eugene M Lukanidin, Noona S Ambartsumian

**Affiliations:** 1 Danish Centre for Translational Breast Cancer Research, Strandboulevarden 49, DK-2100 Copenhagen, Denmark; 2 Department of Molecular Cancer Biology, Institute of Cancer Biology, Danish Cancer Society, Strandboulevarden 49, DK-2100 Copenhagen, Denmark; 3 Department of Proteomics in Cancer, Institute of Cancer Biology, Danish Cancer Society, Strandboulevarden 49, DK-2100 Copenhagen, Denmark

## Abstract

**Introduction:**

Tumour phenotype is regulated in a complex fashion as a result of interactions between malignant cells and the tumour stroma. Fibroblasts are the most abundant and perhaps most active part of the tumour stroma. A better understanding of the changes that occur in fibroblasts in response to the presence of malignant cells may lead to the development of new strategies for cancer treatment. We explored the effects of fibroblasts on the growth and invasion of mammary carcinoma tumour cells *in vitro *and *in vivo*.

**Methods:**

In order to analyse secreted factors that affect invasive abilities of breast cancer cells we co-cultured human mammary fibroblasts (HMF3s) and cancer cells (MCF7S1) in three-dimensional (3D) growth conditions devoid of heterogeneous cell-cell contact. To study the possible influence of fibroblasts on MCF7S1 cancer cell growth *in vivo *we co-injected HMF3s and MCF7S1 cells in Balb/c nu/nu mice.

**Results:**

In 3D co-culture both HMF3s and MCF7S1 cells demonstrated enhanced invasion into a Matrigel matrix. This was correlated with enhanced expression of the metastasis promoting S100A4 protein in fibroblasts, stimulation of the matrix metalloproteinase (MMP)-2 activity, and enhanced secretion of a range of different cytokines. Orthotopic injection of oestrogen-dependent MCF7S1 cancer cells together with fibroblasts showed stimulation of tumour growth in mice without an external oestrogen supply. The resulting tumours were characterized by increased development of extracellular matrix, as well as an increase of murine S100A4 concentration and activity of MMP-2 in the tumour interstitial fluid.

**Conclusion:**

Stimulation of the invasive phenotype of tumour cells in 3D co-cultures with fibroblasts could be correlated with increased production of S100A4 and MMP-2. We propose that enhanced development of mouse host-derived tumour stroma in a MCF7S1 co-injection xenograft model leads to oestrogen independency and is triggered by the initial presence of human fibroblasts.

## Background

In most human tumours, the stroma microenvironment is heavily altered compared with the stroma of normal tissue [1]. Both the composition of the extracellular matrix (ECM) and the ratio between the different cell types present in the microenvironment are different in normal compared with activated stroma [2]. Stroma cells are now well known to play a pivotal role in promoting tumour growth [3,4]. The general consensus is that the stroma triggers neoplastic progression through signals within the stroma environment (reviewed in [5,6]).

The stroma closely associated with benign as well as malignant epithelia consists of ECM and cellular components, including fibroblasts, adipocytes, endothelial and immune cells, all of which have the potential to influence progression of tumour cells toward a more aggressive state [5,7]. Fibroblasts are the most studied stroma cell, and their influence on cancer development has been repeatedly proven [8].

Progression of breast cancer is accompanied with alterations in gene expression both in epithelial cancer cells and cells composing tumour stroma [9]. Alterations in gene expression are at least in part determined by soluble factors produced into the tumour microenvironment both by tumour cells and stroma fibroblasts [10].

Several molecules produced by the stroma cells into the tumour microenvironment are known to stimulate tumour progression. Among these are MMPs [11], different cytokines [12] and the metastasis-associated protein S100A4 [13].

A large number of models have been proposed to study the tumour microenvironment, and significant developments have occurred in the complexity of these models making them more comparable to the *in vivo *models [14,15]. The most commonly used 3D models for include spontaneous cell aggregation, liquid overlay cultures, spinner flask spheroid cultures, and various scaffold-based cultures [16].

To study the effects of stroma components on tumourigenesis various co-culture models involving benign or cancer cells and mostly fibroblasts have been implemented. Krause and colleagues co-cultured the benign breast epithelial MCF10-A cell line with normal mammary fibroblasts to study the importance of stroma in mammary gland development and observed the formation of ductal as well as alveolar structures, both resembling those found *in vivo *[17]. Sadlonova and colleagues used a three-dimensional (3D) co-culture model with epithelial cancer cells and primary fibroblasts and discovered that normal mammary gland-associated fibroblasts were able to inhibit tumour cell proliferation, whereas carcinoma-associated fibroblasts tended to stimulate their growth [18].

In this study using a 3D co-culture system we attempted to identify a panel of soluble factors produced by fibroblasts that stimulate invasion of tumour cells. We also attempted to compare the response of tumour cells to fibroblasts using model systems of different levels of complexity: co-cultured in 2D or 3D *in-vitro*, or grafted to the mammary fat pad of immunodeficient mice.

## Methods

### Cell lines and growth conditions

The BJ fibroblast cell line (established from normal human foreskin) was obtained from the American Type Culture Collection (Rockville, MD, USA). The HMF3s fibroblast cell line, established from healthy mammary tissue [19], was a gift from Professor Mike O'Hare (Ludwig Institute, London, UK). The breast cancer cell lines MCF7 and MCF7S1 (a highly tumour-necrosis factor (TNF)-α sensitive derivative of MCF7), were gifts from Marja Jäättela (Apoptosis Laboratory, Danish Cancer Society, Denmark) [20].

All cell lines were grown in Dulbecco's Modified Eagle's Medium (DMEM) supplemented with 10% fetal calf serum (FCS), penicillin (200 U/ml), and streptomycin sulphate (100 U/ml) in a humidified incubator supplied with 5% CO_2_. Conditioned media (CM) were produced by growing an appropriate cell line in a T75 flask in 15 ml of fresh growth media for 48 h followed by sterile filtration (0.45 μm filter). Cells treated with CM were analysed after 48 h of culturing and were subsequently collected for Western blot analysis using lysis buffer (50 nM Tris-Cl, pH 6.8, 2% sodium dodecyl sulphate [SDS], 0.1% bromophenol blue, 10% glycerol, and 100 mM dithiothreitol). Cell lysate samples for Western analysis were normalised to the total protein content determined by scanning of a Coomassie brilliant blue R-250 stained SDS-PAGE gels. The treatment of cells with CM was repeated four times.

### Measurement of cell length and scattering

The length of fibroblasts treated with standard growth media or tumour cell CM was determined by manual measurement in the Fujifilm MultiGauge software allowing for measurement of irregular shaped cell lengths.

The proportion of scattered tumour cells in the populations treated with DMEM or fibroblast CM was determined by manual classification. Tumour cells were classified as scattered when a visual absence of cell-cell contact was observed.

6 randomly selected fields at 100× magnification were selected for analysis for each cell type in all setups. Each field contained in average 60 fibroblasts or 530 tumour cells to be included in the analysis.

### Mice for in vivo experiments

Intact female Balb/c nu/nu mice were injected with 4 × 10^6 ^cells per cell line in the R2 mammary fat. 10 animals were used per group and selected groups were supplied with oestrogen in the drinking water 6 days prior to the injection of cells (0.67 μg/ml). This was continued for the entire experiment. Tumours were manually measured throughout the experiment in two dimensions, and the volume was calculated using the ellipsoid formula: volume = 1/2 * a * b^2 ^(a = length, b = width) [21]. Animals were sacrificed at a sign of illness or after 100 days at termination of the experiment. All animal experiments were approved by the Animal Welfare Inspectorate at the Danish Ministry of Justice (ref. 2007/561-1395).

All animals with a palpable mass that upon histological examination of random tumour sections did not contain cancer cells were discarded from the analysis and the observed growth was considered an inflammatory response to the injection.

### Tumour interstitial fluid (TIF) analysis

TIFs were produced by cutting the tumours into smaller 1 mm-sized pieces followed by 2 h incubation at 37°C in phosphate-buffered saline (PBS; 1:20 w/v ratio) as originally described by Celis *et al*., 2004 [22]. Prior to analysis, the samples were normalized to the total protein content measured by OD_280_.

### Immunofluorescent cytostaining

Cells were fixed for 20 min in 4% paraformaldehyde followed by membrane permeabilisation with 0.2% Triton X-100 for 2 min. The following primary antibodies were incubated for 1 h at RT: Vimentin (NeoMarker clone Ab-2, 1:1000 dilution), S100A4 (isolated as described in [23], 1:1500 dilution), oestrogen receptor (ER; NeoMarker clone SP1, 1:500 dilution), E-cadherin (Abcam clone decma-1, 1:800 dilution), and pan-cytokeratins (DAKO #Z0622, 1:500 dilution). Alexa Fluor 488 goat anti-rabbit and Alexa Fluor 568 goat anti-mouse were used as secondary antibodies (1:1000 dilution for 1 h incubation at RT). Cells were nuclear stained with 4',6-diamidino-2-phenylindole (DAPI) following mounting with ProLong^® ^Gold antifading media (Invitrogen). Fluorescent images were acquired with a Zeiss AxioImager confocal microscope, and photographs were processed using Zeiss Zen2008 software.

### Immunohistochemical analysis of mouse tissue

Formalin-fixed paraffin-embedded tissue sections were immunostained ON with the following antibodies: ER (NeoMarker clone SP1, 1:500 dilution), progesterone receptor (PgR) (NeoMarker clone SP2, 1:500 dilution), Laminin (Sigma #L-0303, 1:200 dilution), Fibronectin (NeoMarker #RB-077-A0, 1:500 dilution), S100A4 pAb [23] 1:2000 dilution, F4/80 (Accurate Chemicals clone A3-1, 1:2000 dilution) and prolyl-4-hydroxylase (DAKO #M0877, 1:400 dilutions). Immunostaining was performed according to the protocol of the manufacturer. The EnVision^+ ^horseradish peroxidase (HRP)-labeled detection system (DAKO) was utilized as the detection system. For staining with F4/80 antibody, the tyramide signal amplification biotin system was used for detection (PerkinElmer). All slides were counterstained with Mayers hematoxylin.

In order to quantify the proportion of PgR positive cells, 10 random fields at 200× magnification per section were analysed resulting in classification of 1500 cells per tumour in average.

### 3D invasion assay

The experimental setup for this assay is described in detail in Ambartsumian *et al*., 2006 [24]. Briefly, cells were incubated overnight to form clumps on an inverted lid in hanging droplets containing 4 × 10^4 ^or 6.5 × 10^4 ^cells (fibroblasts and cancer cells, respectively) suspended in normal growth media. The formed cell aggregates were manually transferred to a layer of polymerized Matrigel™ (growth factor-reduced, BD) mixed 1:1 with serum-free DMEM prior to use. The aggregate was covered with a sealing layer of Matrigel mix and incubated at 37°C for polymerization after which DMEM with 10% FCS and pen/strep supplements was added. The extent of outgrowth was followed for 5-6 days, and media were changed to 0% FCS conditions 24-48 h prior to harvest. The experiments were repeated at least six separate experiments. A neutralizing IL-6 antibody (R&D systems #MAB2061) was added to co-cultures at a concentration of 1 μg/ml. Pure IL-6 (Sigma, I-1394) in concentrations of 20-50 ng/ml was added to mono-cultures of cancer cells. Experiments were repeated four times.

### Western blot analysis

Proteins were detected using a standard Western blot procedure after separation by SDS-polyacrylamide electrophoresis. Primary antibodies were incubated ON according to the suppliers' instructions: MMP-2 (NeoMarker Ab-2, 1:600 dilution), MMP-9 (NeoMarker clone Ab-5, 1:400 dilution), and affinity purified rabbit anti-S100A4 [23] (1:2000 dilution). HRP-conjugated rabbit anti-mouse or goat anti-rabbit antibodies (DAKO diluted at 1:2000) and ECL-plus chemiluminescent substrate (Amersham) were used for visualisation.

### MMP detection by zymography

Zymography gels were produced from standard 12% SDS-acrylamide gels containing gelatine or β-casein protein at a concentration of 1.2 mg/ml. The gels were pre-run before loading of serum-free CM and run overnight at 4°C at 50 V. Gels were briefly washed and incubated at 37°C overnight for gel degradation (washing buffers: 2 × 30 min with 50 mM Tris-HCl pH 7.5, 2.5% Triton X-100 and 2 × 10 min in 50 mM Tris-HCl pH 7.5. Incubation buffer: 50 mM Tris-HCl Ph 7.5, 0.15 M NaCl, 10 mM CaCl_2_, 0.1% Triton X-100, 0.02% NaN_3_). Gels were stained in Coomassie brilliant blue R-250 solution and subsequently destained in 10% acetic acid. An LAS-1000 analyser and the MultiGauge software from Fuji Film were used to process the images.

### Cytokine detection using pre-probed membranes

A standard ratio of the volume of media to the number of cells was used to produce CM in both 2D and 3D culture experiments enabling us to compare them for cytokine production.

Membrane-based cytokine arrays from RayBiotech (C series 1000, detecting 120 and 96 different human [array VI and VII] and mouse [array III and IV] cytokines, respectively) were used to analyse CM from the 3D model and mouse TIFs. The protocol supplied by the manufacturer was followed with slight modifications. Membranes were blocked for 30 min with protein-free blocking buffer (ThermoScientific), and 1 ml sample was added per membrane and incubated overnight at 4°C. Biotin-conjugated secondary antibodies and HRP-conjugated streptavidin supplied in the kit were both diluted in the protein-free blocking buffer and incubated with membranes for 2 h at room temperature before detection using Amersham ECL™ Advance (Amersham). Spot intensities were quantified by Image Quant TL software by normalization to the background and positive controls.

### Enzyme-linked immunosorbent assay (ELISA)

Sandwich ELISAs specific for murine S100A4 were performed according to the assay previously described by Ambartsumian *et al*., 2001 [25] to measure the concentration of S100A4 protein in mouse TIFs.

### Statistical analyses

Statistical analyses were performed using GraphPad Prism statistical software. Student's t-test was used for evaluation of fibroblast length measurements, tumour cell scattering and differences in protein expression. Immunohistochemical staining of PgR was analysed by Mann-Whitney *t*-test in the GraphPad Prism software.

## Results

### Soluble factors promote morphological changes in cancer cells and fibroblasts

**Figure 1:**
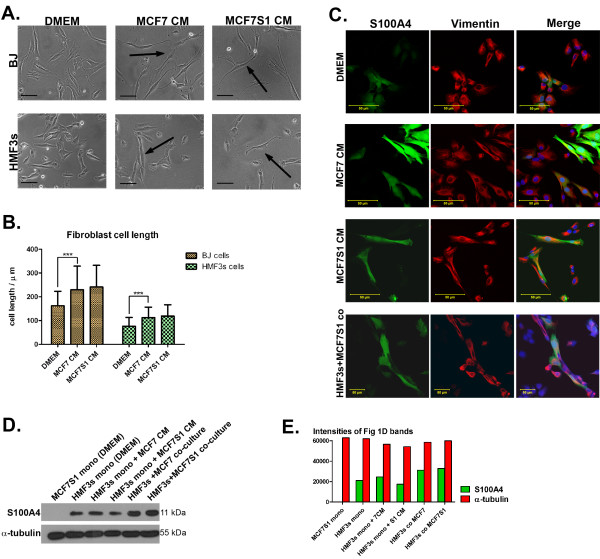
**Characterisation of fibroblasts and the effect of conditioned media from cancer cells**. (A) Phase contrast images of BJ and HMF3s fibroblasts treated with cancer cell CM in 2D growth conditions. Arrows indicate markedly elongated cells. Scale bars = 100 μm. (B) The length of fibroblasts in normal growth conditions and when stimulated with cancer cell CM (*** p < 0,0001 by Student's t-test). (C) Upregulation of S100A4 expression (green) in HMF3s fibroblasts in response to treatment with cancer cell CM or in co-culture with MCF7S1 tumour cells. Vimentin (red) was used as a fibroblast-specific marker. Scale bars = 50 μm. (D) Western blot analysis of cell lysates showing increased S100A4 expression in HMF3s cells in response to co-culturing with tumour cells. A-tubulin was used as a loading control. (E) Quantification of the blot shown in Figure 1D indicating the relative levels of both α-tubulin and S100A4.

To study the effect of fibroblasts on breast cancer cells, we co-cultured human mammary (HMF3s) or foreskin-derived (BJ) fibroblast cell lines with two variants of the oestrogen-dependent breast cancer cell line, MCF7 and its derivative MCF7S1. Alternatively we cultivated these cells in the CM from tumour cells and fibroblasts respectively. Exposure of both fibroblast cell lines to CM conditioned for 48 h by tumour cells induced significant cell elongation (Fig. 1A). Both cancer cell lines produced factors that stimulate elongation of fibroblasts, but the MCF7S1 cells exhibited a more pronounced effect. Calculation of the increase in cell length showed that MCF7S1 CM induced a 49.3% and 55.8% increase in cell length of BJ and HMF3s fibroblasts respectively, whereas CM from MCF7 cells induced elongation by 42.0% (BJ) and 47.7% (HMF3s) respectively (Fig. 1B).

A similar elongating effect was observed when fibroblasts were co-cultured with tumour cells (Fig. 1C, lower panel). Elongation of HMF3s fibroblasts was accompanied by increased S100A4 expression, which is known to be expressed in activated fibroblasts [23] (Fig. 1C). Western blot analysis of cell lysates obtained after treatment of cells with CM from cancer cells, as well as from co-culturing of fibroblasts with cancer cells, confirmed this observation (Fig. 1D).

**Figure 2:**
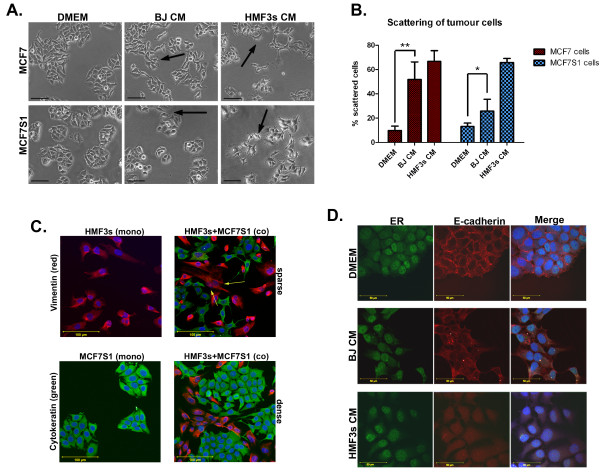
**Characterisation of cancer cells and the effect of conditioned media from fibroblasts**. (A) Phase contrast images of MCF7 and MCF7S1 cells treated with fibroblast CM in 2D growth conditions. Arrows indicate scattered cancer cells. Scale bars = 100 μm. (B) Percentage of cancer cells scattered in normal growth conditions and in response to fibroblast CM (** p = 0,0006, * p = 0,0225 by Student's t-test). (C) Elongation of HMF3s fibroblasts (arrows) and scattering of MCF7S1 cells in sparse co-culture using vimentin (red) and cytokeratin (green) as cell-specific markers. Mono-cultures of both cell lines were used as controls. In dense co-culture, fibroblasts shorten and tumour cells are less scattered. Scale bars = 100 μm. (D) Downregulation of E-cadherin expression (red) in MCF7S1 cells in response to treatment with fibroblast CM. Oestrogen receptor (ER) (green) was used as a marker for cancer cells. Scale bars = 50 μm.

Conversely, exposure of tumour cells to CM from fibroblasts stimulated scattering of the cells which otherwise grew in characteristic clusters (Fig. 2A, left). The arrows in Fig. 2A show examples of highly scattered cells detached from clusters and with protrusions on the cell surface. Calculation of the proportion of scattered cells showed that cancer cells were more responsive to HMF3s CM (Fig. 2B) compared to both BJ CM and DMEM growth media. These results suggest that orthotopic factors from the mammary fibroblasts affect breast cancer cells differently than those derived from skin. Fig. 2C shows cell-specific staining of HMF3s fibroblasts and MCF7S1 cancer cells against vimentin and cytokeratins, respectively. These markers are used to distinguish cell types in co-cultures (Fig. 2C, right panel) showing fibroblast elongation and cancer cell scattering compared with the mono-cultures (Fig. 2C, two images in left panel). Cancer cells reorganise into clusters separated by streaks of fibroblasts when the co-cultures become denser (Fig. 2C, lower right panel). Immunofluorescent staining demonstrated that morphological changes of tumour cells were accompanied by the decrease of E-cadherin, a hallmark of the epithelial-mesenchymal transition (EMT) [26] (Fig. 2D). The data indicate that soluble factors produced by fibroblasts induce an EMT-like state in cancer cells. Conversely, factors produced by cancer cells induce morphological changes in fibroblasts.

### Co-culturing fibroblasts and breast cancer cells in 3D Matrigel stimulates invasion of cancer cells

**Figure 3:**
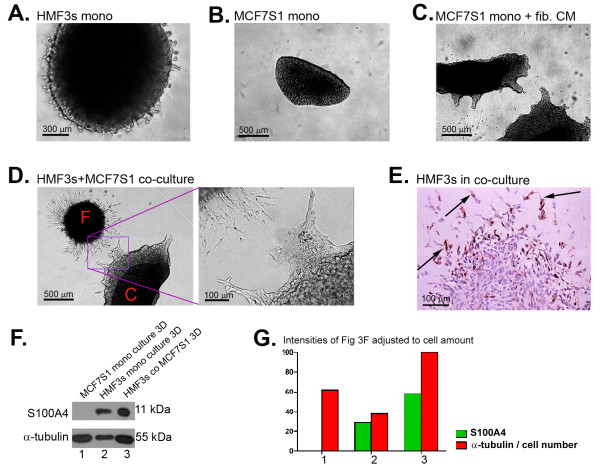
**Invasion of fibroblasts and cancer cells in 3D culturing conditions**. (A) Phase contrast images of HMF3s fibroblasts grown in Matrigel in mono-culture. Notice sparse outgrowth of HMF3s cells. Scale bar = 300 μm. (B) Phase contrast images of MCF7S1 tumour cells grown in Matrigel in mono-culture. Notice absence of invasion in MCF7S1 cancer cells. Scale bar = 500 μm. (C) Invasion of MCF7S1 cancer cells in response to treatment with fibroblast CM. Notice pronounced invasion of cancer cells. Scale bar = 500 μm. (D) Mutual invasion of HMF3s fibroblasts [F] and MCF7S1 cancer cells [C] co-cultured in 3D Matrigel. Inset shows details of the invasive front. Scale bars = 500 and 100 μm, respectively. (E) Immunohistochemical detection of S100A4 in the invasive front of HMF3s fibroblasts grown in co-culture with MCF7S1 cells. Arrows point to invading cells. Scale bar = 100 μm. (F) Western blot analysis of cell lysates from 3D cultures showing increased S100A4 expression co-cultures. α-tubulin was used as a loading control. Note that the expected amount of α-tubulin in lane 3 is equal to the sum of lane 1 and 2. (G) Quantification of the blot shown in Figure 3F with tubulin adjusted to the cell amounts in each lane. The co-culture (3) represents 100%.

To investigate whether soluble factors produced by fibroblasts are able to induce changes in tumour cells, we studied the behaviour of tumour cells and fibroblasts grown in 3D Matrigel as a support matrix without direct contact between the two cell types. This was achieved by generating individual fibroblast and tumour cell aggregates which were placed into a thick Matrigel layer separate from each other (at a distance of 0.5-2 mm). MCF7S1 and HMF3s cells exhibit a stronger mutual response when grown under conventional 2D culturing conditions; therefore, we chose these two cell lines for further analysis. The MDA-MB-231 and ZR-75-1 cell lines were also tested in the 3D Matrigel model and gave similar results (data not shown). Neither HMF3s nor MCF7S1 cell aggregates showed any signs of invasive growth when grown in mono-cultures (Fig. 3A and 3B). In contrast, co-cultures of MCF7S1 and HMF3s cells demonstrated clear signs of invasive growth into the Matrigel (Fig. 3D). In most cases, we observed a clear tendency toward site-directed growth. Fibroblast CM also stimulated MCF7S1 cells to invade the gel, which again confirms the importance of secreted factors (Fig. 3C). Co-culture of fibroblasts with MCF7S1 cells also led to expression of S100A4 at the leading edge of the fibroblast aggregate (Fig. 3E). This observation was confirmed by Western blot analysis of cell lysates obtained from 3D cultures (Fig. 3F), where a 2-fold upregulation of S100A4 was observed in 3D co-cultures compared to fibroblasts in mono-cultures (green bars in Fig. 3G). MCF7S1 cells did not express the S100A4 protein.

### 3D culturing stimulates secretion of cytokines

**Figure 4:**
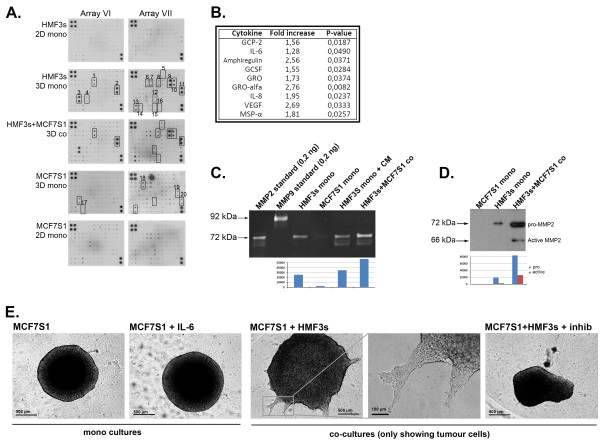
**Cytokine microarray and zymography analysis of CM from 2D and 3D Matrigel model**. (A) Comparison of cytokine microarray membranes from different growth conditions of HMF3s and MCF7S1 cells. The cytokines altered in 3D culturing conditions in HMF3s or MCF7S1 cells are numbered and correspond to: 1, GCP-2; 2, IL-6; 3, MCP-1; 4, MCP-3; 5, Amphiregulin; 6, Fas/TNFRSF6; 7, FGF-4; 8, GCSF; 9, GRO; 10, GRO-α; 11, IL-8; 12, MSP-α; 13: TIMP-1, 14, TIMP-2; 15, uPAR (urokinase plasminogen activator receptor); 16, VEGF (vascular-endothelial growth factor); 17, SDF-1; 18, ENA-78; 19, IL-6R; 20, sTNF-RI. (B) Significant increase in cytokine levels in CM from 3D Matrigel co-culture of HMF3s and MCF7S1 cancer cells compared with 3D mono-cultures (upregulated cytokines are marked on the membranes from co-culture). (C) Gelatine zymography showing upregulation of MMP-2 activity. The lower panel shows quantification of band intensities. (D) Western blot analysis of CM from 3D Matrigel co-culture of HMF3s and MCF7S1 cell aggregates showing marked upregulation of MMP-2. The lower panel shows quantification of band intensities of both pro- and active-MMP2. (E) 3D invasion assay stimulated with IL-6 in MCF7S1 monocultures and inhibited with IL-6-specific inhibitor in MCF7S1 + HMF3s co-cultures. A co-culture without the inhibitor is shown for reference.

The ability of fibroblast CM to stimulate the invasion of tumour cells into the 3D matrix indicated that secreted factors produced by these cells in response to the 3D culture conditions may be sufficient to induce the observed invasiveness of tumour cells. Therefore, we analysed a range of cytokines produced by tumour cells and fibroblasts grown in different culture conditions (Fig. 4A). The cytokine protein microarray, which is able to detect 120 different cytokines, showed that mono-cultures of tumour cells and fibroblasts are induced to produce increased amounts of cytokines when cultured in 3D Matrigel compared with traditional 2D conditions (Fig. 4A, numbered cytokines in 3D mono cultures). This was especially clear with HMF3s fibroblasts, in which upregulation of cytokines, such as granulocyte chemotactic peptide (GCP)-2, interleukin (IL)-6, IL-8, tissue inhibitor of metalloproteinases (TIMP), growth-related oncogene (GRO), and monocyte chemoattractant protein (MCP)-1, was documented. MCF7S1 cells upregulated the production of MCP-1, TIMP1, epithelial neutrophil activating protein (ENA)-78, and IL-6R and downregulated stroma-derived factor (SDF)-1 and macrophage-stimulating protein (MSP)-α in 3D growth. Co-culturing of tumour cells and fibroblasts in 3D conditions did not result in the appearance of new cytokines; rather, the amounts of certain cytokines already upregulated in 3D mono-cultures, such as GCP-2, IL-6, amphiregulin, GRO/GRO-α, IL8, and MSP-α, were further increased (Fig. 4B). Additional data on differential expression of cytokines is shown in the Additional file 1.

To test the input of identified cytokines in stimulation of invasion we chose IL-6 because of its well known association to tumour progression [27]. IL-6 was added to tumour cells in 3D cultures but we failed to observe any stimulation of tumour cell invasion (Fig. 4E). In contrast to that, addition of a neutralizing anti-IL-6 antibody completely abolished the stimulation of tumour cell invasion induced by fibroblasts in co-cultures. This indicates that IL-6 is essential but not sufficient alone to stimulate fibroblast induced invasion of tumour cells.

The IL-6 antibody had no effect on invasion of fibroblasts in the co-culture (data not shown).

Although the shift from 2D to 3D growth conditions of fibroblasts and tumour cells led to a dramatic increase in cytokine production, such a change did not result in the invasive behaviour of these cells in mono-culture, whereas co-culturing of these cells markedly stimulated invasion of both cell types.

### 3D co-cultures induce MMP2 production

One of the functions of stroma fibroblasts is to produce ECM-degrading enzymes, thereby stimulating cancer cell invasion [28]. Consequently, we hypothesized that the invasive growth we observed in 3D co-cultures could be mediated by activation of matrix-degrading proteinases. The CM obtained from 3D co-cultures was therefore analysed for the presence of protease activity by gelatine zymography. CM from MCF7S1 mono-cultures did not contain any detectable proteolytic activity, whereas CM from HMF3s mono-cultures exhibited gelatinolytic activity to match the MMP-2 standard of 72 kDa (Fig. 4C). Casein-based zymographies were also performed but generally showed only very little activity (data not shown). Co-cultured cells revealed intensification of the 72 kDa bands as well as the appearance of lower molecular weight bands (Fig. 4B). We propose that these lower molecular weight bands correspond to the active form of MMP2. To verify the upregulation of MMP-2, we performed Western blot analysis of CM from 3D mono- and co-cultures with MMP-2-specific antibodies, confirming MMP-2 as the activated protease (Fig. 4D). Gelatine zymography was also performed with CM from mono-and co-cultures of HMF3s and MCF7S1 cells grown in 2D conditions. Although CM from HMF3s cells in 2D contained MMP-2 protease activity, it was only slightly upregulated upon co-culturing with MCF7S1 cells compared to the 3D co-culture conditions, and no additional bands appeared in 2D (data not shown).

### Fibroblasts stimulate tumour growth in mouse xenografts via activation of mouse stroma

Our data demonstrated that factors produced by fibroblasts stimulated invasion of MCF7S1 tumour cells in 3D Matrigel. We therefore proposed that fibroblasts may be able to affect MCF7S1 tumour growth in an orthotopic mouse model. MCF7S1 cells grafted into mice require oestrogen to maintain growth [29]; oestrogen-independent growth of these cells is an indicator of their progressed state [30]. To test whether the presence of fibroblasts could render MCF7S1 cells ability to grow in the absence of oestrogen, MSF7S1 cells in combination with HMF3s were orthotopically implanted into mammary fat pads of Balb/c nu/nu mice with and without oestrogen supplementation.

**Figure 5:**
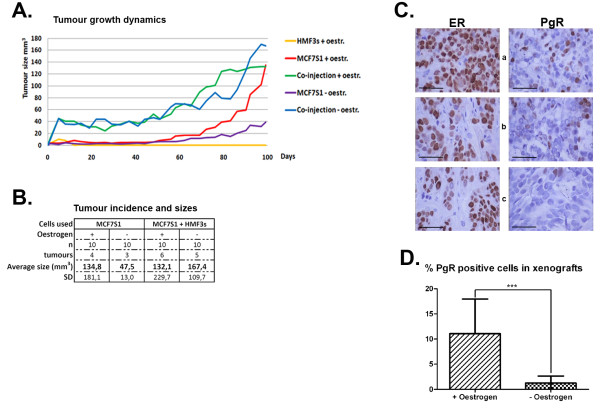
**HMF3s fibroblasts stimulate tumour growth of MCF7S1 cells in xenograft mouse model**. (A) Dynamics of tumour growth in mice implanted with MCF7S1 + HMF3s cells with and without oestrogen supplementation. (B) Tumour incidence and average tumour sizes in different experimental groups. (C) Immunohistochemical staining of tumour sections with ER- and PgR-specific antibodies. The mice were injected with (a) MCF7S1 cells + oestrogen, (b) MCFS7S1 + HMF3s cells +oestrogen. (c) MCFS7S1 + HMF3s cells without oestrogen. Scale bars = 50 μm. Notice downregulation of PgR expression in the tumour sections without hormone supplementation. PgR expression is almost absent in xenografts without the hormone supplementation. (D) Quantification of PgR-positive cells in tumour xenografts with and without hormone supplementation (*** p = 0,0006).

The dynamics of tumour growth were monitored for 100 days. We were able to document the steady growth of tumours when MCF7S1 cells were implanted together with HMF3s fibroblasts, both with and without oestrogen supplementation (Fig. 5A). In the presence of oestrogen, HMF3s fibroblasts provided MCF7S1 cells with additional growth capabilities by the acceleration of tumour growth initiation as compared to the tumours developed without HMF3s cells (Fig. 5A). Fig. 5B shows that the average sizes of tumours developed in mice injected with MCF7S1 + HMF3s cells were similar at the time of termination of the experiment independent of the presence of supplemented oestrogen. At post mortem analysis, tissue sections obtained from the regions with palpable tumour-like mass detected in the control group injected with MSF7S1 cells without estrogen supplementation (Fig 5B) revealed absence of tumour cells.

We were not able to detect metastasis in the lungs of mice from any experimental groups.

We next performed immunohistochemical staining of tumour sections with antibodies against ER and PgR (Fig. 5C). ER expression was similar in the tumour sections obtained from mice from all experimental groups. In contrast, tumours that developed in mice implanted with MCF7S1 + HMF3s cells without oestrogen showed significant downregulation of PgR, a well-known downstream target of ER [31] (Fig. 5C and 5D). Downregulation of PgR expression therefore indicated that at the time of sacrifice, the levels of oestrogen, endogenous or produced by the grafted cells, at the site of implantation were below the concentration required to induce PgR expression. We therefore assumed that the growth of MSF7S1 cells co-injected with HMF3s fibroblasts was supported by the production of other factors rather than increased oestrogen production in the mouse.

**Figure 6:**
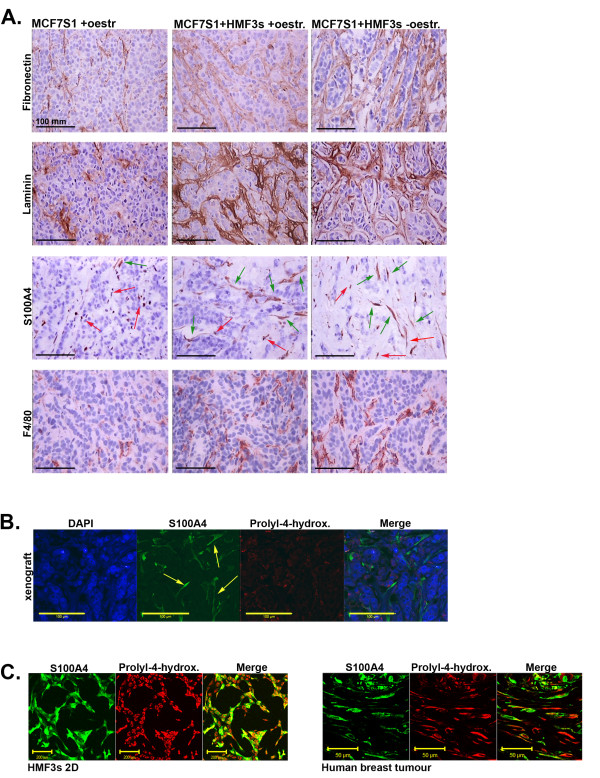
**Immunohistochemical analysis of tumour xenografts**. (A) Immunohistochemical staining of tumour xenografts against fibronectin, laminin, S100A4, and F4/80. Green arrows indicate elongated S100A4-positive cells, and red arrows indicate smaller rounded cells. Scale bars = 100 μm. (B) Double-immunostaining of tumour sections from HMF3s + MCF7S1 co-injections detecting S100A4 (green) and human prolyl-4-hydroxylase (red). Yellow arrows indicate elongated cells expressing S100A4 but not prolyl-4-hydroxylase. Scale bar = 100 μm. (C) Positive control for prolyl-4-hydroxylase staining on cell cultures of the HMF3s fibroblast (red = prolyl-4-hydr, S100A4 = green, scale bar = 200 μm) and a human tumour sample (scale bar = 50 μm).

We attempted to detect the presence of HMF3s human fibroblasts in MCF7S1 + HMF3s tumours. Immunostaining of tumour sections with human fibroblast-specific prolyl-4-hydroxylase antibodies [32] did not reveal the presence of any positive cells (Fig. 6B). HMF3s fibroblasts stained positive for prolyl-4-hydroxylase in cell cultures (Fig. 6C, left) and the marker also detects fibroblasts in human breast tumour tissue (Fig. 6C, right).

Immunostaining of tumour sections with anti-S100A4 polyclonal antibodies that recognise both human and murine S100A4 revealed the presence of numerous S100A4-positive cells in the stroma. Some of these cells had an elongated morphology (notice green arrows in Fig. 6A and yellow arrows in Fig. 6B), and we presume that these were mouse fibroblasts replacing human cells in the course of tumour development. S100A4-positive cells were increased in MCF7S1 + HMF3s tumours compared with MCF7S1 tumours (Fig. 6A). Other S100A4-positive cells had morphologically rounded features and were likely to be immune cells (red arrows in Fig. 6A).

Immunohistochemical staining with mouse macrophage-specific F4/80 antibodies showed that mouse macrophages were much more abundant in MCF7S1 + HMF3s tumours compared with MCF7S1 tumours (Fig. 6A, lower panel), suggesting that augmented development of mouse-derived stroma produces soluble factors that support oestrogen-independent growth of MCF7S1 tumour cells.

Immunohistochemical staining of tumour sections for the components of ECM; fibronectin and laminin, showed that in contrast to MCF7S1 tumours, MCF7S1 + HMF3s tumours contained an extremely well-developed ECM (Fig. 6A).

All this indicates that the tumour stroma of MCF7S1 + HMF3s tumours was much more developed.

### Co-injection of human fibroblasts stimulates the production of murine S100A4 and MMP2

**Figure 7:**
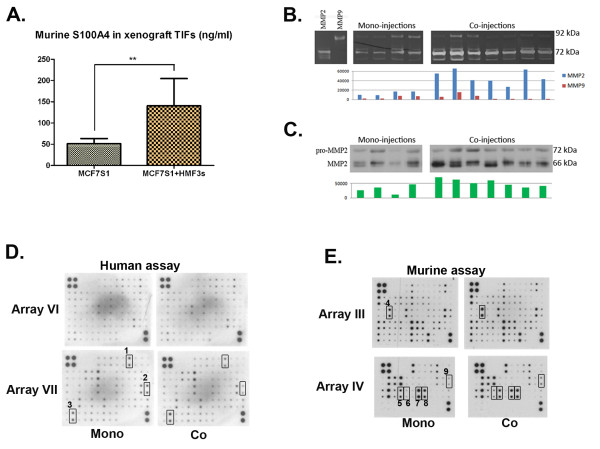
**Analysis of TIFs from tumour xenograft tumours**. (A) Concentration of murine S100A4 in TIFs measured by mouse-specific sandwich ELISA (** *p *= 0,001). (B) Gelatine zymography showing upregulation of MMP-2 activity in TIFs obtained from tumours co-injected with HMF3s and MCF7S1 cells. The lower panel shows quantification of the activity of MMP-2 and MMP-9. Notice that only MMP-2 activity is significantly upregulated (*p *= 0.001). (C) Western blot analysis of TIFs from tumours co-injected with HMF3s and MCF7S1 cells showing upregulation of MMP-2 expression. The lower panel shows quantification of band intensities with a significant increase (*p *= 0.029). (D) Comparison of human-specific cytokine profiles of TIFs from mono- and co-injection xenografts. Cytokines altered from mono- to co-injections are numbered 1 to 3 (1, bFGF↓; 2, IL-8↓; 3, TIMP-1↑). (D) Comparison of mouse-specific cytokine profiles of TIFs from mono- and co-injection xenografts. Cytokines altered from mono- to co-injections are numbered 4 to 9 (4, IL-6↑; 5, pro-MMP9↓; 6, Resistin↑; 7, Thymus CK-1↓; 8, TIMP-2↑; 9, MMP2↑).

The metastasis-related protein S100A4, which is known to be produced by stroma cells, has been linked to both angiogenesis and the invasion of tumour cells as an extracellular factor [25,33]. Therefore, we investigated whether increased numbers of observed S100A4-positive stroma cells correlated with its secretion by analysing TIFs using an S100A4 mouse-specific sandwich ELISA. Fig. 7A shows an increased concentration of S100A4 in TIFs from MCF7S1 + HMF3s tumours compared with MCF7S1 tumours. The murine S100A4 concentration in TIFs from mice injected with cancer cells alone ranged from 39.4 to 60.3 ng/ml (mean, 51.5 ± 12.2 ng/ml). TIFs from MCF7S1 + HMF3s ranged from 60.0 to 241.3 ng/ml (mean, 161.5 ± 62.0 ng/ml), resulting in a significant increase in S100A4 release (*p *= 0.01). The average murine S100A4 concentration in TIFs from MCF7S1 + HMF3s tumours was 2.5-3-fold higher than in TIFs from MCF7S1 tumours. TIFs were also investigated using a human-specific sandwich ELISA assay, but no human S100A4 was detected.

We next investigated protease activity present in TIFs from MCF7S1 and MCF7S1 + HMF3s tumours. Gelatin zymography revealed several major and minor bands, of which two coincided with the MMP-2 and MMP-9 standards (Fig. 7B, upper panel). Quantification of the intensity of the MMP-2-specific bands showed increased activity of MMP-2 in TIFs isolated from MCF7S1 + HMF3s tumours (Fig. 7B, lower panel). MMP-2 upregulation was confirmed by Western blot analysis with MMP-2-specific antibodies (Fig. 7C). MMP-9 activity in co-injection xenografts was not found to be different from mono-injections.

We observed an enhancement of cytokine release in the 3D model in co-culture conditions, and we compared this cytokine release in tumours to determine whether the fibroblasts were also able to stimulate cytokine production *in vivo*. Human-specific cytokine microarray analysis revealed that TIFs from MCF7S1 or MCF7S1 + HMF3s tumours generally contained low levels of human cytokines compared with the 3D model (Fig. 7D). TIFs from MCF7S1 + HMF3s tumours contained slightly lower levels of IL-8 and basic fibroblast growth factor (bFGF) and increased levels of TIMP-1 (cytokine numbers 1, 2, and 3, respectively).

In contrast, a similar analysis performed using a mouse-specific cytokine microarray revealed that TIFs from MCF7S1 + HMF3s xenografts contained increased amounts of numerous mouse-specific cytokines, in particular resistin, IL-6, and MMP-2 (Fig. 7E, cytokine no. 6, 4, and 9, respectively). The level of pro-MMP-9 was lowered in TIFs from co-injection xenografts (Fig. 7E, cytokine no. 5).

We assume, therefore, that stimulation of MCF7S1 cell growth triggered by human fibroblasts in an orthotopic mouse model is dependent on the pronounced development of host-derived stroma that supply tumour growth by the mouse-derived cytokines, S100A4 and MMPs.

## Discussion

Despite the increasing appreciation of the importance of tumour-stroma interactions in the progression of cancer, little is known about the factors regulating the crosstalk between stroma and neoplastic cells. Fibroblasts represent the major cellular component of cancer-associated stroma [34,35]. Although their role in accelerating cancer growth and possibly causing malignant conversion has been demonstrated, the molecular factors regulating these processes remain largely unknown [36].

A number of studies have suggested that stroma-associated fibroblasts exert their stimulatory effect by undergoing genetic changes [37,38]. It has been shown that genetic alteration in the stroma is a more accurate predictor of prognosis than whole tissue signatures [39,40]. However, other mechanisms operating at the level of (de)regulation of gene expression may be more important during the early stages of the disease when rapid and massive changes are evoked in the stroma.

We attempted to analyse the reciprocal changes that occur in tumour cells and fibroblasts by modelling tumour-stroma interactions, with a main focus on soluble factors able to stimulate tumour progression and produced by human mammary fibroblast. We compared three model systems in which MCF7S1 breast cancer cells and HMF3s human mammary fibroblasts were co-cultured in 2D conditions and 3D Matrigel and orthotopically injected *in vivo *into the mouse mammary fat pad.

In 2D culture conditions fibroblast CM induced scattering of both MCF7 and MCF7S1 cells with subsequent downregulation of E-cadherin. Loss of E-cadherin expression has been correlated with *in vitro *invasiveness and *in vivo *tumour progression [41]. Downregulation of E-cadherin in our system was correlated with changes in tumour cell morphology but not with changes of vimentin or α-smooth muscle actin expression (data not shown). EMT is a complex multistage process that includes morphological changes accompanied by modulation of the expression of numerous proteins, in particular cytoskeletal and adhesion molecules [42]. We therefore propose that at short term co-culturing of tumour cells with fibroblasts we observe early steps of EMT-like changes that can eventually lead to appearance of a mesenchymal phenotype [43]. Simultaneously, CM from cancer cells upregulated S100A4 expression, which is known to be overexpressed in activated fibroblasts and HMF3s fibroblasts, and stimulated substantial elongation of fibroblasts. These observations point to the role of soluble factors produced by tumour cells and fibroblasts in stimulation of morphological changes in both cell types.

Several groups have demonstrated that normal fibroblasts exert an inhibitory effect on tumour cells, whereas the tumour-associated fibroblasts stimulate tumour cell growth and proliferation [18,44]. Immortalized fibroblast cell lines used in this study express smooth-musle actin and S100A4, pointing to their activated state [45].

To analyse the input of soluble factors on the growth of tumour cells and fibroblasts in 3D culture conditions we cultivated MCF7S1 and HMF3s cell aggregates in 3D Matrigel in the absence of direct contact between cells. Both cell types were induced to invade the ECM when grown in co-culture, but not in mono-culture. Again, we obtained evidence demonstrating that the presence of soluble factors was sufficient to stimulate invasion without the need for direct cell-cell contact.

3D culturing conditions led to a dramatic increase in release of a number of cytokines. The most prominent cytokines that have also been shown to be linked with cancer development were IL-6 [27,46], IL-8 (CXCL8) [47,48], GRO/GRO-α (CXCL1) [49,50], GCP-2 (CXCL6) [51], and TIMP-1 [52,53]. These cytokines were virtually not detectable in CM from 2D growth and were highly upregulated in CM from 3D Matrigel mono-cultures with HMF3s cells.

Co-culturing of fibroblasts with tumour cells in 3D Matrigel led to further upregulation of many of these cytokines. This was especially pronounced with GCP-2, IL-6, IL-8, GRO/GRO-α, and MSP-α. Interestingly, increased expression of the identified cytokines in the tumour microenvironment has been associated with poor prognosis in many cancer types [54,55].

Invasion of tumour cells by addition of a single cytokine (IL-6) was not enough to trigger the complex process of tumour cell invasion. Whereas blocking of IL-6 by a neutralising antibody showed that increased amounts of this cytokine was necessary for tumour cell invasion, indicating that a complex cytokine network is needed for stimulation of this process.

In addition to cytokine activation 3D co-culturing also stimulated increased production of matrix metalloproteinases, in particular MMP2. MMP-2 is known to be induced in tumour stroma cells, mainly by tumour-associated fibroblasts in response to the presence of tumour cells or tumour-produced factors [56,57].

Similar to the 2D co-culture conditions expression of S100A4 in HMF3s fibroblasts was also up-regulated in 3D co-culture. S100A4 is a stromal derived factor [45] capable of stimulating the production of several MMPs [58,59] and induce angiogenesis by attraction of endothelial cells [25]. Furthermore, T-cells are attracted to the tumour milieu by S100A4 and thereby inducing an inflammatory response in the tumour microenvironment through elevation of T-cell specific cytokines [60].

Altogether these observations indicate that a 3D co-culturing model leads to an increase in the production of a number of molecules that are capable of stimulating aggressive invasive behaviour of cancer cells. In the 3D co-culture model used in the present work, the invasive phenotype was induced only by soluble factors, because cell aggregates were co-cultured in 3D Matrigel in the absence of direct contact. This contrasts with other 3D co-culture models that have been developed and used to study reciprocal direct cellular interactions between tumour and stroma cells [44,61,62].

We suggest that the 3D co-culture model used in the present study could be useful for studying soluble factors produced in the tumour microenvironment by certain combinations of tumour and stroma cells.

Factors that were up-regulated in 3D co-cultures of breast cancer cells and fibroblasts have been shown to be involved in malignant progression of breast cancer. It has been shown that elevated serum levels of IL-6 and MMP-2 in breast cancer patients correlates with the stage and the severity of the disease [63,64]. MMP-2 is expressed in the early stages of breast cancer and is believed to contribute to the first events leading to tumour formation because of its ability to degrade the basement membrane [65].

The observed increase in S100A4 in our xenograft model is of great interest since it has been shown in several studies how overexpression of this protein is correlated with a poor prognostic outcome in breast cancer patients [66,67].

To assess the stimulatory effect of activated fibroblasts on tumour cells in *in vivo *conditions we orthotopically injected MSF7S1 cells in combination with HMF3s fibroblasts into the immunodeficient mice with and without oestrogen supplementation. Growth of ER-positive MCF7S1 cells is oestrogen-dependent, both *in vitro *and *in vivo*, in mouse xenograft models [29].

Orthotopic injection of these cells with HMF3s human fibroblasts into the mammary fat pad of immunodeficient mice led to the formation of tumours in the absence of oestrogen supplementation which indicated that MSF7S1 cells acquired a more aggressive phenotype.

Tumours were characterized by extensive development of stroma. Our analysis revealed that the stroma formed in these tumours did not contain human-specific fibroblasts. Moreover, we were not able to detect an increase in the production of human-specific cytokines released in TIFs of MCF7S1 + HMF3s tumours.

Mouse-specific cytokine antibody array analysis of MSF7S1 + HMF3s tumours showed that they contain increased numbers of overexpressed cytokines compared with TIFs generated from MCF7S1 tumours. The most prominent increase was observed in the levels of resistin, which has been identified as a biomarker of bladder cancer [68] and is up-regulated in women with polycystic ovarian syndrome [69] but has not been linked directly with mammary cancer.

MMP-2 was upregulated in co-injection tumours, demonstrated by zymography, Western blot, and cytokine array analysis of TIF. The upregulation of murine cytokines correlated with increased infiltration of murine cells into human xenografts.

The expression of S100A4 has been previously shown to be stimulated by growth factors [70].

We documented an increase in the secretion of murine, but not human S100A4 into the MCF7S1 + HMF3s tumour microenvironment. The S100A4 protein is produced by murine fibroblasts and macrophages [71,72] which were found in larger numbers in tumour xenografts from co-injections. It has been shown that S100A4 in xenograft injection experiments can render MCF7 cells oestrogen independent [73]. We propose that the observed increase in S100A4 is one of the factors responsible for the oestrogen independent growth of MCF7S1 + HMF3s xenografts.

It is possible that another cytokine upregulated in the MCF7S1 + HMF3s tumours alone or in combination could trigger hormone-independent growth of MCF7S1 cells. It has been shown previously that cytokines, such as VEGF, FGF, TGF-β and BMP could stimulate hormone-independent growth of MCF7 cells in vivo [74-78].

Human fibroblasts have been shown previously to stimulate tumour progression in orthotopic mouse models [44,79], and human stroma cells are shown to be replaced by mouse host-derived stroma cells in a similar orthotopic transplantation model [80].

We therefore speculate that human fibroblasts perform the initial stimulation of MCF7S1 tumour cell growth, possibly by producing increased oestrogen concentrations [81] and by attracting murine stroma cells to infiltrate the growing tumour and create a microenvironment that support hormone-independent growth of MCF7S1 tumour cells.

## Conclusion

Co-culturing of human tumour cells and fibroblasts in 3D Matrigel in the absence of direct cell contacts stimulates the production of a complex network of molecules that induce invasion of both cell types generating a more aggressive phenotype. Moreover, the mouse model experiments suggest that the murine host-derived cytokine network replaces human-derived cytokines in stimulation of hormone-independent growth of MCF7S1 breast cancer cells.

## Competing interests

The authors declare that they have no competing interests.

## Authors' contributions

CJO carried out all experimental work of the study, designed the graphic presentation of the results, and participated in drafting the manuscript. JM participated in the design of the study and contributed to drafting the manuscript. EL contributed to drafting the manuscript. NA participated in the design of the study and participated in drafting the manuscript. All authors read and approved the final manuscript.

## Pre-publication history

The pre-publication history for this paper can be accessed here:

http://www.biomedcentral.com/1471-2407/10/444/prepub

## Supplementary Material

Additional file 1 **Complete table of quantification of cytokine detection arrays**. Quantification of spot intensities from RayBiotech cytokine detection arrays including the calculated fold increase and p-values for each cytokine. The cytokines marked in red are significantly increased in co-culture compared to the mono-cultures combined and are emphasised in Fig. 4B. Click here for file
