# Supplementary material for: Human mammary fibroblasts stimulate invasion of breast cancer cells in a three-dimensional culture and increase stroma development in mouse xenografts
Source: BMC Cancer. 2010 Aug 19;10:444. doi: 10.1186/1471-2407-10-444 (PMC2933628; doi:10.1186/1471-2407-10-444)
Supplement: Additional file 1 — Complete table of quantification of cytokine detection arrays. Quantification of spot intensities from RayBiotech cytokine detection arrays including the calculated fold increase and p-values for each cytokine. The cytokines marked in red are significantly increased in co-culture compared to the mono-cultures combined and are emphasised in Fig. 4B. [file 1471-2407-10-444-S1.PDF]

Supplementary data - table 1

	HMF3s mono	3 co S1	MCF7S1 mono	Fold increase	p-value
IL-5	3312,5	4370,2	n.d.	1,32	0,1146
IL-6	27624,0	36568,3	1145,2	1,28	0,0490
MCP-1	20983,0	25415,6	4580,0	0,99	0,7127
LIGHT - very low	745,0	1556,8	462,5	1,29	0,1246
RANTES	1440,5	2622,8	3670,5	0,51	0,0033
BDNF	2040,5	2932,2	n.d.	1,44	0,1510
GCP-2	7272,5	11335,1	n.d.	1,56	0,0187
EGF	2554,5	2855,5	n.d.	1,12	0,6212
SDF-1	4009,5	4681,8	n.d.	1,17	0,5356
AgRP	2750,7	2781,8	n.d.	1,01	0,9661
Angiopoietin-2	3668,0	5274,9	2178,4	0,90	0,2966
Amphiregulin	2513,9	8921,7	964,8	2,56	0,0371
Axl	2540,1	3843,1	n.d.	1,51	0,6073
Fas/TNFRSF6	5520,6	9127,4	2791,5	1,10	0,3645
FGF-4	9368,8	13612,4	5493,9	0,92	0,1320
FGF-9	5396,6	8013,2	1827,2	1,11	0,1818
GCSF	8640,5	13404,0	n.d.	1,55	0,0284
GITR-Ligand	6738,6	8198,8	510,6	1,13	0,0879
GRO	22567,8	38957,1	n.d.	1,73	0,0374
GRO-α	10745,1	29673,7	n.d.	2,76	0,0082
IL-8	21276,6	41479,7	n.d.	1,95	0,0237
IGFBP-3	1933,4	4202,2	1027,0	1,42	0,1943
IGFBP-6	3132,7	6828,6	n.d.	2,18	0,0632
TIMP-1	11983,3	22806,6	n.d.	1,90	0,0652
TIMP-2	6357,8	11836,9	120,7	1,83	0,1988
Thrombopoietin	2181,5	5029,7	n.d.	2,31	0,1102
TRAIL R3	2265,1	3397,6	570,0	1,20	0,4341
TRAIL R4	4344,2	5362,0	1329,0	0,95	0,6705
uPAR	3363,2	9094,6	812,0	2,18	0,2491
VEGF	3314,6	10918,3	744,0	2,69	0,0333
VEGF-D	2207,8	4748,3	1197,0	1,39	0,1393
Lymphotactin	6081,1	8178,0	2373,1	0,97	0,2899
MIF	5462,7	7063,1	2445,7	0,89	0,1188
MIP-1α	5316,5	8117,1	2109,6	1,09	0,2577
MIP-1β	7591,5	8496,9	2873,9	0,81	0,7781
MSP-α	8104,3	21605,2	3845,2	1,81	0,0257
Osteoprotegerin	3992,6	7119,4	2194,5	1,15	0,2672
sTNF-RI	2139,3	4650,5	5202,5	0,63	0,1317

n.d. - Signal was equal to background levels, and no readout was obtained.
A fold increase less than 1 indicates a down-regulation.
Readouts less than 5000 were not considered reliable for quantification
